# The beginning of a seed: regulatory mechanisms of double fertilization

**DOI:** 10.3389/fpls.2014.00452

**Published:** 2014-09-11

**Authors:** Andrea Bleckmann, Svenja Alter, Thomas Dresselhaus

**Affiliations:** ^1^Cell Biology and Plant Biochemistry, Biochemie-Zentrum Regensburg, University of RegensburgRegensburg, Germany; ^2^Plant Breeding, Center of Life and Food Sciences Weihenstephan, Technische Universität MünchenFreising, Germany

**Keywords:** pollen tube, ovule, gamete interaction, cell fusion, signaling, fertilization, polyspermy

## Abstract

The launch of seed development in flowering plants (angiosperms) is initiated by the process of double fertilization: two male gametes (sperm cells) fuse with two female gametes (egg and central cell) to form the precursor cells of the two major seed components, the embryo and endosperm, respectively. The immobile sperm cells are delivered by the pollen tube toward the ovule harboring the female gametophyte by species-specific pollen tube guidance and attraction mechanisms. After pollen tube burst inside the female gametophyte, the two sperm cells fuse with the egg and central cell initiating seed development. The fertilized central cell forms the endosperm while the fertilized egg cell, the zygote, will form the actual embryo and suspensor. The latter structure connects the embryo with the sporophytic maternal tissues of the developing seed. The underlying mechanisms of double fertilization are tightly regulated to ensure delivery of functional sperm cells and the formation of both, a functional zygote and endosperm. In this review we will discuss the current state of knowledge about the processes of directed pollen tube growth and its communication with the synergid cells resulting in pollen tube burst, the interaction of the four gametes leading to cell fusion and finally discuss mechanisms how flowering plants prevent multiple sperm cell entry (polyspermy) to maximize their reproductive success.

## Introduction

**Figure 1:**
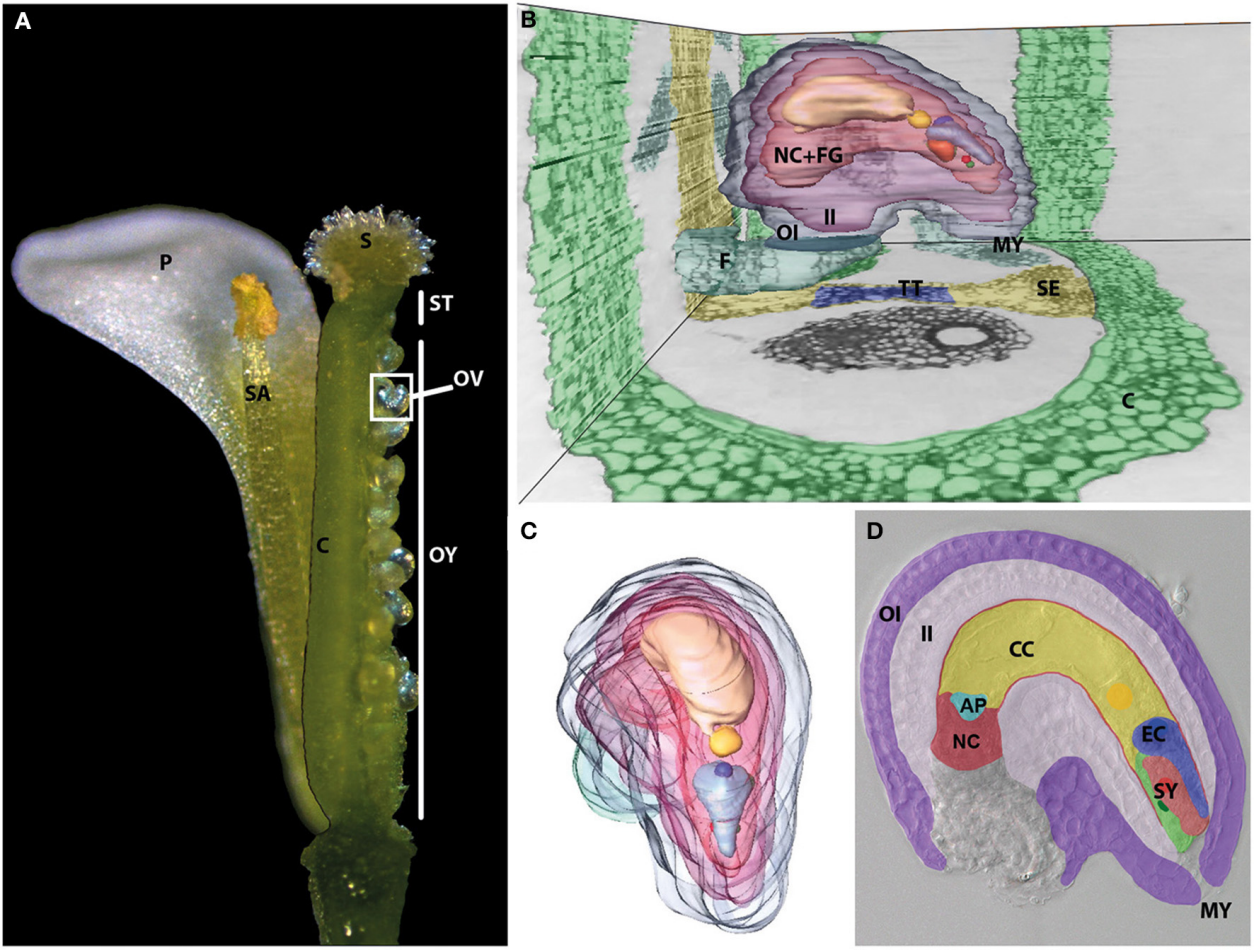
**The female gametophyte is deeply imbedded inside the female flower organs**. **(A)** Dissected and reconstructed *Arabidopsis* flower. One of four petals (P) and one of six stamina (SA) are shown. They surround the pistil, which represents the female flower organ. It can be dissected into three parts. The upper part contains the papilla cells and forms the stigma (S), which is connected to the ovary (OY) by the style (ST). The ovary is formed by two fused carpels (C), which harbor two rows of ovules (OV). A side view **(B)** and front view **(C)** of a 3D-remodeled ovule reconstructed from toluidine blue stained single, successive ultra-thin sections of a dissected pistil. See Supplemental Movie 1 for whole series of sections. The ovule is connected to the septum (SE, yellow) containing the transmitting tract (TT, blue) by the funiculus (F, petrol) and surrounded by the carpel tissue (C) (green). A 3D-model of a dissected ovule shown from various angles is shown in Supplemental Movie 2. The mature female gametophyte cells (FG) and the nucellus tissue (NC) are surrounded by the outer (OI) and inner integuments (II) (OI, blue; II, purple). The vacuole and nucleus of the different female gametophyte cells showed highest contrast and are therefore shown individually. Near to the micropyle (MY), the two nuclei of the two synergid cells (SY) are shown in red and green. The egg cell, indicated by EC in **(D)**, has a comparably large vacuole (light blue) and its nucleus (blue) is located at its chalazal pole. The center of the female gametophyte is filled by the vacuole (light yellow) of the central cell, indicated by CC in **(D)**, and its homo-diploid nucleus (yellow). The three degenerating antipodal cells, indicated by AP in turquoise color in **(D)** at the chalazal pole are not highlighted. **(D)** DIC microscopic image of a mature female gametophyte surrounded by the maternal sporophytic tissues of the ovule. The cell types and tissues are artificially colored as shown in **(B,C)**. At full maturity the nucellus cell (NC) layer surrounding the developing embryo sac is flattened between inner integument (II) and female gametophyte cells.

High crop yield strongly depends on efficient formation of numerous ovules, which after successful fertilization, develop into seeds comprising seed coat, embryo, and endosperm. In angiosperms, the haploid gametophytic generations produce the male and female gametes required to execute double fertilization. Both gametophytes are reduced to only a few cells. The female gametophyte is deeply embedded and thus protected by the maternal sporophytic tissues of the pistil (Figure 1). It harbors the female gametes (egg and central cell) and is surrounded by the nucellus tissue as well as the inner and outer integuments. After fertilization these different tissues form the seed coat. The female gametophyte arises from a megaspore mother cell though processes known as megasporogenesis and megagametogenesis (for review see Evans and Grossniklaus, 2009; Drews and Koltunow, 2011). In ~70% of all angiosperm species including *Arabidopsis* and maize the embryo sac develops according to the Polygonum type (Drews et al., 1998). The functional megaspore undergoes three mitotic divisions resulting in a syncytium containing eight nuclei. After nuclei migration and cellularization seven cells are differentiated: the haploid egg cell and its two adjoining synergid cells are located at the micropylar pole forming the egg apparatus. The homodiploid central cell containing two fused or attached nuclei is located more centrally, whereas three antipodal cells are found at the chalazal pole of the ovule opposite to the egg apparatus. While synergid cells are essential for pollen tube attraction, burst and sperm cell release (see below), the function of antipodal cells is so far unknown. During female gametophyte maturation antipodal cells are degenerating in the ovule of the eudicot model plant *Arabidopsis* (Mansfield et al., 1991), whereas they proliferate in other species including grasses and form a cluster of about 20–40 cells (Diboll and Larson, 1966).

The haploid male gametophyte (pollen grain) is formed during the processes of microsporogenesis and microgametogenesis from the microspore mother cell by meiosis and two successive mitotic divisions resulting in the formation of a tricellulate pollen grain. The vegetative cell encases the two sperm cells, which are connected with the vegetative cell nucleus by the generative cell plasma membrane, forming the male germ unit (MGU). MGU formation ensures the simultaneous delivery of both gametes to the ovule (for review see McCue et al., 2011). The major task of the vegetative cell is to deliver the sperm cells through the maternal tissues of the style and ovary to an unfertilized ovule. After pollen germination, the vegetative cell forms a tube and grows by tip-based-growth mechanism along papillae cells of the stigma into the style toward the transmitting tract. Inside the transmitting tract, pollen tubes are guided toward the ovules by mechanical and chemotactic cues involving numerous interactions with the sporophytic style tissues. In many eudicots pollen tubes exit the transmitting tract and grow along the septum, the funiculus and the outer integument toward the micropyle of unfertilized ovules. In grasses the ovary contains a single ovule and the pollen tube is directly guided toward its surface after leaving the blind ending transmitting tract. The pollen tube continues to grow along its surface toward the micropylar region (for review see Lausser and Dresselhaus, 2010). Finally, the pollen tube enters the micropyle, an opening between the inner and outer integuments, and grows toward the two synergid cells. The pollen tube bursts and sperm cells are released. This process is associated with the degeneration of the receptive synergid cell due to programmed cell death. Subsequently, both sperm cells arrive at the gamete fusion site and fertilize the egg and central cell (Hamamura et al., 2011). From the moment of germination until sperm discharge the pollen grain/tube communicates with at least five different sporophytic and three different gametophytic cell types to successfully accomplish fertilization (Palanivelu and Tsukamoto, 2012). Its extended growth inside the female flower tissue is regulated by many different guidance, attraction and support mechanisms. After sperm cell release all gametes are activated, followed by fusion of their membranes and nuclei by processes known as plasma- and karyogamy, respectively. After successful double fertilization further signaling events are activated to prevent polyspermy. In this review we will summarize and discuss the cell–cell communication processes, which are essential to successfully accomplish double fertilization and to initiate seed development in angiosperms.

## Pollen tube growth and attraction

### Pollen rejection

Pollen tube growth and guidance toward the female gametes are controlled at various stages by chemotactic signals and growth support molecules derived from the sporophytic and gametophytic tissues of the female flower organs. Pollen grains placed on the stigma (Figure 1A) by contact, wind or different pollinators stick to the papilla cells and start to hydrate followed by their germination. The efficient adhesion of the pollen grain to the papilla cell is regulated by interaction events between these cells and may activate thereby inter- and intra-species barriers to prevent unsuccessful pollination and fertilization events already at this early time point during reproduction. Angiosperms possess different strategies to recognize self from alien pollen and evolved independent self-incompatibility (SI) mechanisms to prevent self-fertilization. Early SI mechanisms are based on cell-cell communication events between the papilla cells and the pollen grains, whereas later SI mechanisms occur while the growing pollen tube interacts with the cells of the transmitting tract. Species of the *Solanaceae*, for example, use a pistil-expressed S-RNase, which penetrates the pollen tube (McClure et al., 1989; Luu et al., 2000). A compatible pollen tube expresses the S-locus F-box protein (SLF), which leads to the degradation of the S-RNase (Hua and Kao, 2006; Kubo et al., 2010), while in incompatible interactions intact S-RNase degrades RNAs resulting, for example, in the disruption of the actin cytoskeleton and other cellular processes (Liu et al., 2007; Roldán et al., 2012). In *Papaveraceae* SI depends on the small pistil secreted protein *Papaver rhoeas* style S (PrsS), which binds to the S-locus pollen tube membrane protein *P. r*. pollen S (PrpS) and activates a Ca^2+^-dependent signaling cascade resulting in pollen inhibition and programmed cell death (Wheeler et al., 2009; Wu et al., 2011). SI is best understood in *Brassicaceae*, which use a surface-localized S-locus receptor kinase (SRK) in papilla cells (Takasaki et al., 2000) and a pollen coat localized cysteine-rich protein (SP11/SCR) (Schopfer, 1999; Shiba et al., 2001) to distinguish self from alien pollen. Their successful interaction leads to proteasome dependent degradation of Exo70A1, an essential component of the exocyst complex. It is thought to be involved in secretion of essential pollen germination factors necessary for pollen hydration (Synek et al., 2006; Samuel et al., 2009). Rejection of pollen in *Brassicaceae* thus occurs already during pollen hydration and germination at the surface of papilla cells. Little is known about SI in the economically important grasses (reviewed in Dresselhaus et al., 2011). Pollen hydration and germination appear not to be affected, although only grass pollen tubes are capable of penetrating the style and reach the transmitting tract. This indicates that SI in the grasses depends on successful interaction of the pollen tube with the sporophytic cells of the style and transmitting tract. The signaling events involved in this recognition process still await their discovery. More details about SI mechanisms can be found in Iwano and Takayama (2012), Watanabe et al. (2012) and Dresselhaus and Franklin-Tong (2013).

### Pollen tube guidance toward and through the transmitting tract

After adhesion and hydration, compatible pollen germinates, penetrates the style and grows through the extracellular space of stylar cells toward the transmitting tract (Figure 1B). The growth direction of the pollen tube is regulated by the formation of different gradients including water, γ-amino butyric acid (GABA), calcium and other small molecules such as D-serine. The water flow during hydration forms an external gradient specifying the site of pollen tube outgrowth and was shown to be controlled by triacylglyceride (Lush et al., 1998; Wolters-Arts et al., 1998). Ca^2+^ influx into the pollen tube tip region is known to be essential for germination and tube growth (Brewbaker and Kwack, 1963; for a review see Steinhorst and Kudla, 2013a) and leads to the generation of an oscillating apex-based cytoplasmic Ca^2+^ (Ca^2+^_cyto_) gradient (Miller et al., 1992; Calder et al., 1997). Initially, papilla cells export Ca^2+^_cyto_ by the auto-inhibited Ca^2+^-ATPase13 (ACA13) at the pollen grain adhesion site (Iwano et al., 2004, 2014). Extracellular Ca^2+^ is then imported into the pollen tube by glutamate receptor-like channels (GLRs), which can be stimulated by D-serine (Michard et al., 2011). In animal systems it was shown that GLRs are non-selective cation channels catalyzing Na^+^ and/or Ca^2+^ influx into cells. Binding of the agonist D-serine to GLRs should thus lead to channel opening resulting in a Ca^2+^_cyto_ increase (Gilliham et al., 2006). D-serine is produced by Serine-Racemase1 (SR1), which shows an expression peak in the style indicating D-serine availability. The induced changes in Ca^2+^_cyto_ concentration in the pollen tube might thereafter regulate and coordinate many different signaling events like actin polymerization and thus influence pollen tube growth behavior and growth direction. Ca^2+^_cyto_-sensors, belonging to the protein families of calmodulin (CaM), calmodulin-like proteins (CMLs), calcium-dependent protein kinases (CDPKs), and calcineurin B-like proteins (CBLs) are expressed in the pollen tube and are thought to control different cell-cell communication events indicated by their localization and overexpression phenotypes. The presence of these different Ca^2+^_cyto_ receptors around the sperm cells and at the pollen tube tip indicate an essential role of Ca^2+^ signals both during pollen tube growth and double fertilization (Zhou et al., 2009; Steinhorst and Kudla, 2013b).

During pollen tube growth the tip needs to modulate the surrounding cell wall of stylar cells enabling its penetration through the extra-cellular space, most likely by interaction with extensin-like proteins and arabinogalactan proteins as well as the secretion of cell wall softening enzymes and inhibitors such as polygalacturonases and pectin methylesterase inhibitors (Cosgrove et al., 1997; Grobe et al., 1999; Stratford et al., 2001; Ogawa et al., 2009; Nguema-Ona et al., 2012; Woriedh et al., 2013). The transmitting tract is composed of small cylindrical cells that are surrounded by an extracellular matrix (ECM), which contains a mixture of glycoproteins, glycolipids, and polysaccharides (Lennon et al., 1998). The ECM provides essential nutrients as well as components for an accelerated, extended and guided pollen tube growth (Palanivelu and Preuss, 2006). Without an intact transmitting tract like in the *NO TRANSMITTING TRACT* (*NTT*) mutant or its target *HALF FILLED* (*HAF*), pollen tube growth is severely affected and either slowed down or prematurely terminated. *NTT* encodes a C2H2/C2HC zinc finger transcription factor involved in ECM production and is essential for programmed cell death in the transmitting tract upon pollination (Crawford et al., 2007). *HAF* encodes a bHLH transcription factor and is involved in NTT dependent transmitting tract regulation (Crawford and Yanofsky, 2011). The transmitting tract-specific arabinogalactan glycoproteins TTS1 and TTS2 have a positive effect on *in vitro* grown tobacco pollen and show a gradient of increased glycosylation correlating with pollen tube growth direction inside the transmitting tract (Cheung et al., 1995; Wu et al., 1995). Another factor which has a positive effect on pollen tube growth and guidance is chemocyanin, a small secreted peptide in the style of lily (Kim et al., 2003). The different sporophyte-derived signals do not only guide or increase pollen tube growth rate, but rather lead to a change in the pollen transcriptome and thereby activate the pollen for female gametophyte-derived attraction signals (Higashiyama et al., 1998; Palanivelu and Preuss, 2006). Recently, *de novo* expression of closely related MYB transcription factors and other genes were reported to be induced during pollen tube growth through the style regulating themselves a number of downstream genes. Hence pollen tubes maturate during their growth through the sporophytic tissue and thereby become competent for fertilization (Leydon et al., 2013, 2014).

### Ovular pollen tube guidance

The signaling events that control pollen tube exit from the transmitting tract and guidance toward the ovule are not known. In *Arabidopsis* this process was shown to be tightly regulated and usually only a single pollen tube exits the transmitting tract in proximity of an unfertilized ovule. The pollen tube grows on the septum surface toward the funiculus, the tissue connecting the ovule with the septum (Figures 1B–D; Supplemental Movies 1, 2). At the funiculus the pollen tube is directed through the micropyle inside the ovule by a mechanism known as micropylar guidance (Shimizu and Okada, 2000). In *Arabidopsis* a gradient of GABA was reported in front of the ovule. The transaminase POLLEN ON PISTIL2 (POP2) forms this gradient through GABA degradation. At moderate concentrations GABA stimulates pollen tube growth and thus likely supports growth toward the ovule (Palanivelu et al., 2003). Another candidate involved in micropylar guidance is D-serine, which was already described above. Its synthesizing enzyme gene *SR1* is also expressed in the ovule indicating the presence of D-serine (Michard et al., 2011). Semi-*in vitro* fertilization experiments revealed an oscillation of Ca^2+^_cyto_ levels in growing pollen tubes depending on their distance from an unfertilized ovule and especially from the synergid cells (Shi et al., 2009; Iwano et al., 2012). The connection between Ca^2+^_cyto_ and D-serine by GLR channels in growing pollen tubes was already described above. The observed changes in the Ca^2+^_cyto_ level depending on its distance from the synergid cells might again result from this interplay.

Recently, two pollen-expressed mitogen-activated protein kinases (MAPKs), MPK3 and MPK6, were identified in *Arabidopsis*, which are part of the ovular guidance network. *In vivo* pollination assays revealed that *mpk3/6* double mutant pollen tubes were not capable of growing along the funiculus after transmitting tract exit but micropylar guidance (see below) was not effected in the double mutants (Guan et al., 2014). MPK3/6 are two cytoplasmic protein kinases, which seem to be part of the signaling cascade mediating extracellular stimuli to changes in pollen tube growth direction.

In summary, our current understanding of ovular pollen tube guidance is very limited, but a whole orchestra of small molecules derived from the ovule seem to be involved in pollen tube growth support and attraction, and multiple signaling networks are required in pollen tubes to respond to the diverse set of signals and to direct their growth behavior.

### Micropylar pollen tube guidance

**Figure 2:**
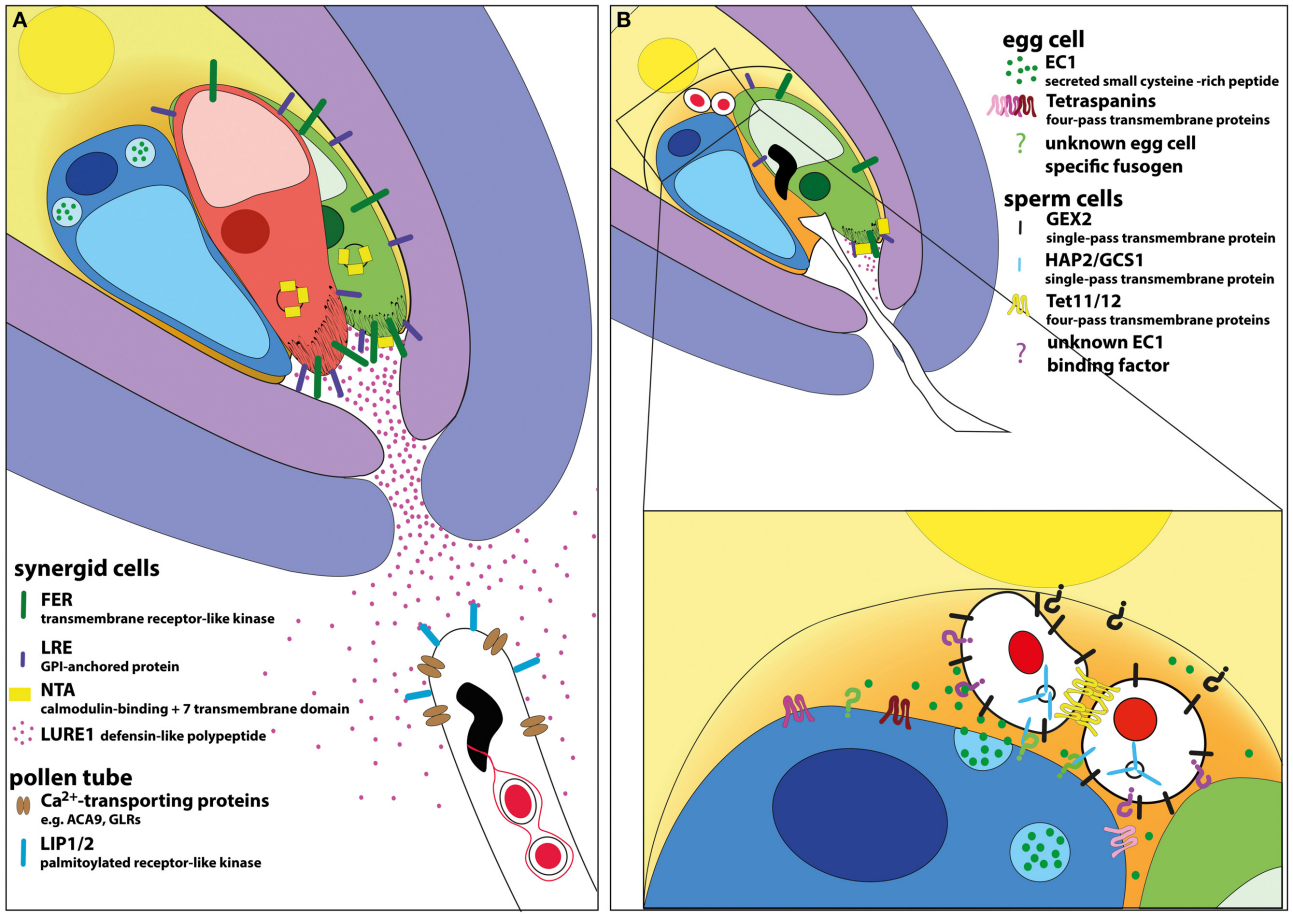
**Model of signaling events during micropylar pollen tube attraction and double fertilization in *Arabidopsis*. (A)** The micropylar opening of the ovule is formed by the inner and outer integuments. The female gametophyte is “naked” at its micropylar pole containing one egg (colored in blue) and two synergid cells (colored in green and red) representing the egg apparatus. The central cell surrounds the egg apparatus. The synergid cells are the main sources of pollen tube attractants. Among other components, they secrete LURE peptides, which bind to pollen expressed LIP1/2 receptors thus directing pollen tube growth. Calcium transporters are involved in pollen tube growth control. The plasma membranes of synergid cells harbor a high concentration of receptors like FER and LRE, especially in the region of the filiform apparatus. Upon pollen tube perception NTA is relocated to the plasma membrane by FER activity likely regulated by Ca^2+^ oscillations inside the synergid cells. **(B)** The pollen tube bursts when it reaches a certain point beyond the filiform apparatus and releases its cytoplasmic contents including the two sperm cells. Pollen tube burst depends on the presence and activation of FER-, LER-, NTA-, and VDD-dependent signaling cascades culminating in the death of the receptive synergid cell (indicated by diffusing red color). Released sperm cells are located at the gamete fusion side, between the two female gametes. The two sperm cells are connected to each other, likely involving tetraspanins. The male gametes adhere to female gametes by GEX2 located at their surface. After activation, the egg cell secrets EC1 leading to sperm cell activation and HAP2/GCS1 localization to the plasma membrane. HAP2/GCS1 and tetraspanins at the surface of gametes may be involved in mediating membrane fusion. Unknown egg and central cell-specific fusogenic proteins as well as EC1 receptor are indicated by question marks in green, black, and purple, respectively.

After arrival at the surface of the ovule, the pollen tube reaches the last phase of its journey, which is known as micropylar pollen tube guidance. It enters the micropyle, an opening between the two integuments, and directly grows toward the egg apparatus in species such as *Arabidopsis* (Figure 2A). In grasses the pollen tube first has to overcome a few layers of nucellus cells (Márton et al., 2005) before it also gets in contact with the filiform apparatus of the synergid cells, a thickened and elaborated cell wall at their micropylar pole, where the cell surface is extensively invaginated (Willemse and van Went, 1984; Huang and Russell, 1992). It was believed for a long time that the pollen tube grows through the filiform apparatus to enter one synergid cell, leading to pollen tube burst and cell death of the receptive synergid cell. Recently, it was shown that the pollen tube is repelled by the filiform apparatus and instead grows along the cell wall of the synergid cells until it reaches a certain point after the filiform apparatus where its growth is arrested and burst occurs explosively (Leshem et al., 2013). Pollen tube burst results in the discharge of its cytoplasmic contents including the two sperm cells. The synergid cells represent the main source for chemo-attractants required for micropylar pollen tube guidance. Moreover, laser ablation experiments in *Torenia fournieri* have demonstrated that a single synergid cell is sufficient and necessary to attract pollen tubes (Higashiyama et al., 2001). The major function of the filiform apparatus may thus be to considerably increase the micropylar surface of the synergid cells, which represent glandular cells of the egg apparatus. Many known components required for pollen tube growth and guidance are membrane-associated and accumulate at the filiform apparatus, which gives it the additional role of a signaling platform. It contains, for example, a high Ca^2+^ concentration, which is known to play a key role during the regulation of pollen tube growth (Brewbaker and Kwack, 1963; Chaubal and Reger, 1990; Iwano et al., 2004; Michard et al., 2011) and also seems to trigger pollen tube burst afterwards (see below). In *Arabidopsis* the formation of the filiform apparatus as well as the expression of different attractants in the synergid cells depend on the activity of the R2R3-type Myb transcription factor MYB98 (Kasahara et al., 2005; Punwani et al., 2007). Among other genes, *MYB98* regulates the expression of genes encoding cysteine-rich proteins (CRPs), including those representing a subgroup of defensin-like (DEFL) polypeptides (Punwani et al., 2008; Takeuchi and Higashiyama, 2012).

In *Torenia* it was shown that a DEFL subgroup of CRPs called LUREs are secreted from the synergid cells and accumulate at the filiform apparatus (Okuda et al., 2009; Kanaoka et al., 2011). LUREs attract pollen tubes in a species-preferential manner from a distance of about 100–150 μm and were recently shown to bind to the tip region of pollen tubes (Okuda et al., 2009, 2013). Due to their rapid molecular evolution it was difficult to identify orthologs in other plant species, but finally the DEFL subgroup CRP810/AtLURE1 of *Arabidopsis* was discovered to be involved in micropylar pollen tube guidance (Takeuchi and Higashiyama, 2012). In *Zea mays*, EGG APPARATUS1 (ZmEA1), a small hydrophobic precursor protein of 94 aa was reported as an egg apparatus-specific protein required for micropylar pollen tube guidance (Márton et al., 2005, 2012). ZmEA1 was shown to bind in a species-specific manner to the apical region of the pollen tube, where it is quickly internalized and degraded, likely keeping the pollen tube susceptible to pollen tube attractants while growing through the micropylar nucellus cell layers (Márton et al., 2012; Uebler et al., 2013).

More puzzling is the role of the central cell in micropylar guidance of the pollen tube. For example *magatama* (*maa*) mutants show defects in central cell maturation; both haploid nuclei are smaller and often fail to fuse. Pollen tubes grow in the direction of an unfertilized *maa* ovule but loose their way just before entering the micropyle. Moreover, mutant female gametophytes attracted two pollen tubes at a high frequency (Shimizu and Okada, 2000). *MAA3* was recently shown to encode a helicase required for general RNA metabolism, which could explain the central cell maturation defect but not the defect in pollen tube guidance (Shimizu et al., 2008). Another example of central cell-dependent defects in micropylar pollen tube guidance is the transcriptional regulator CENTRAL CELL GUIDANCE (CCG), which is expressed exclusively in the central cell of the female gametophyte (Chen et al., 2007). These guidance defects may be indirect and caused by non-functional or immature central cells influencing maturation of egg apparatus cells and thus the generation of guidance components in these cells. It might also be possible that molecules generated by the MAA3 and CCG pathways directly regulate the generation of guidance molecules in the neighboring cells. Also the egg cell seems to be involved in micropylar guidance. GAMETE EXPRESSED 3 (GEX3) is a plasma membrane-localized protein, which is expressed in the unfertilized egg cell. Down-regulation of *GEX3* by antisense RNA in the egg cell leads to defects in micropylar guidance by an unknown mechanism (Alandete-Saez et al., 2008).

Until recently, male factors and signaling pathways reacting to attractants secreted from the egg apparatus were unknown. The receptor-like kinases (RLKs) LOST IN POLLEN TUBE GUIDANCE1 (LIP1) and 2 (LIP2) have been identified, which are preferentially expressed in the pollen tube. Both proteins show membrane localization due to a palmitoylation site and are involved in the AtLURE1-dependent guidance mechanism. *lip1/2* double mutant pollen reach the funiculus but fail to grow through the micropyle inside the ovule, and the pollen tube shows a reduced attraction toward AtLURE1 (Liu et al., 2013). However, it is unclear whether LIP1/2 are directly involved in LURE perception.

## Pollen tube burst and sperm cell discharge

Pollen tube burst seems to be regulated by RLKs located at surfaces of both male and female interaction partners (Figures 2A,B). The RLK FERONIA/SIRENE (FER/SRN) is expressed in most tissues including the synergid cells, where it localizes predominately at their surface in the filiform apparatus region. Loss-of-function mutants display a pollen tube-overgrowth phenotype. Pollen tube growth arrest and sperm cell discharge fail in *fer* ovules (Huck et al., 2003; Rotman et al., 2003; Escobar-Restrepo et al., 2007). *FER* acts as a cell surface regulator for RAC/ROP GTPases. Recently it was shown that FER binds to the small secreted peptide Rapid Alkalinization Factor (RALF), which leads to the inhibition of a plasma membrane H^+^-ATPase resulting in the suppression of cell elongation in the primary root (Haruta et al., 2014). Besides changes in the pH, RALF also induces the increase of Ca^2+^_cyto_ (Pearce et al., 2001; Haruta and Constabel, 2003; Haruta et al., 2008) and thus may influence pollen tube growth arrest and eventually its burst. The *Arabidopsis* genome contains around 30 *RALF*-like genes indicating the possibility that a pollen secreted RALF-like peptide may indeed be involved in FER-dependent pollen tube perception (Olsen et al., 2002). Other proteins were identified whose loss-of-function resemble the *fer* phenotype such as the glycosylphosphatidylinositol (GPI)-anchored protein LORELEI (LRE) and the Mildew Resistance Locus O (MLO) family protein NORTIA (NTA). Both genes are expressed in synergid cells and show a similar pollen tube overgrowth phenotype (Capron et al., 2008; Kessler et al., 2010). *NTA* encodes a protein with multiple potential transmembrane domains as well as a calmodulin-binding site. The frequency of unfertilized ovules in *lre/lre* and *nta/nta* is less pronounced compared to *fer/fer* pistils. This finding indicates that FER activity is essential for pollen tube perception, while other, yet unknown factors act redundantly with LRE and NTA. These factors are all present at the synergid plasma membrane during pollen tube contact. However, while FER accumulates at the filiform apparatus already prior to pollen tube arrival, NTA relocalizes to the plasma membrane of the synergid cells at the filiform apparatus region upon pollen tube contact. In a transient expression system NTA is directly targeted to the plasma membrane. However, in *Arabidopsis* ovules under control of its endogenous promoter, NTA localizes to uncharacterized compartments within the cell, and becomes relocalized to the plasma membrane upon pollen tube arrival, indicating the presence of an active retention mechanism. This relocalization is FER-dependent and therefore connects FER to NTA in the same signaling network (Kessler et al., 2010). The presence of a calmodulin-binding domain in NTA supports the idea of a Ca^2+^-dependent signaling network, which is activated upon pollen tube arrival. Due to its predicted signal peptide and GPI anchor, LRE is expected to localize to the extracellular side of the plasma membrane after passage through the secretory pathway. But so far plasma membrane localization could only be shown in a transient expression system and not in synergid cells themselves (Capron et al., 2008). Whether LRE localization also changes during the process of fertilization needs to be elucidated. Another factor required for successful pollen tube/synergid cell communication is VERDANDI (VDD), a member of the plant-specific B3 superfamily of transcription factors. *Vdd* mutants show defects in antipodal and synergid cell identity and result in the lack of pollen tube burst after reaching the synergid cells. In contrast to *fer, nta*, or *lre* mutants, an overgrowth phenotype was not reported indicating that VDD may act downstream of cell surface signaling components (Matias-Hernandez et al., 2010). Little is known about male factors involved in pollen tube/synergid cell communication. Two closely related homologs of FER, ANXUR1 (ANX1), and ANX2 were reported to be involved in the timing of pollen tube burst or more precisely in the inhibition of pollen tube burst. In an *in vitro* pollen tube growth assay, pollen of double mutants show spontaneous discharge already after pollen bulge formation, whereas *in vivo*-grown pollen tubes germinate normally on a stigma but rupture in the style before arriving at the egg apparatus. Both receptors localize mainly to the apical tip of the pollen tube as well as in small vesicles (Boisson-Dernier et al., 2009; Miyazaki et al., 2009). Their over-expression inhibits growth by over-acting exocytosis and over-accumulation of secreted cell wall material (Boisson-Dernier et al., 2013) suggesting that the main function is associated with coordination of growth through the style rather than sperm cell discharge. Other male factors, which are involved in pollen tube growth and reception, are the pollen-expressed transcription factors MYB97, MYB101 and MYB120. *myb97/101/120* triple mutants exhibited uncontrolled growth and failed to discharge their sperm cells after entering the embryo sac (Liang et al., 2013). It is thought that these factors are required to enable pollen tube to communicate with the pistil tissues and the female gametophyte (Leydon et al., 2014). As already mentioned, the level of Ca^2+^_cyto_ alters during pollen tube elongation. Upon pollen tube arrival Ca^2+^_cyto_ level starts to oscillate in the synergid cells, triggered by the contact of the pollen tube tip with the synergid cell. This oscillation can be observed until pollen tube burst, which leads to the degeneration of one synergid cell (Sandaklie-Nikolova et al., 2007; Iwano et al., 2012; Denninger et al., 2014; Ngo et al., 2014). These changes in Ca^2+^_cyto_ level are essential for sperm delivery and are depending on FER and LRE activity, respectively. Downstream of FER, NTA localization to the synergid cell surface and its activity likely depend on sufficient Ca^2+^_cyto_ level in the synergid cells (Ngo et al., 2014). The synergid cell, which is in contact with the pollen tube follows a regulated cell death program that is somehow associated and controlled by pollen tube burst and linked to oscillation of Ca^2+^_cyto_ level (Higashiyama et al., 2000; Sandaklie-Nikolova et al., 2007; Denninger et al., 2014; Ngo et al., 2014). It cannot be explained by mechanical breakdown due to an invading pollen tube. In *fer* mutant, for example, pollen tube growth continues around the synergid cells, which must be associated with mechanical stress but does not induce synergid cell death (Escobar-Restrepo et al., 2007). However, the signaling events, which are responsible for programmed cell death in the synergid cell, are not understood yet. In maize pollen tube arrival is associated with the secretion of defensin-like ZmES proteins, inducing pollen tube burst by activating the K^+^ -channel *Zea mays* 1 (KZM1) in the pollen tube membrane (Amien et al., 2010). Whether these “toxin”-like molecules are also capable of inducing synergid cell burst remains to be shown.

## Gamete interaction and prevention of polyspermy

### Sperm cell delivery, activation, and gametic membrane interactions

Once released, the two sperm cells are delivered to the so-called gamete fusion site between egg and central cell (Figure 2B). It is controversial whether this requires active transport or is solely based on cytoplasmic flow associated with burst of both pollen tube and receptive synergid cell and/or the architecture of the egg apparatus. Most flowering plants, like *Arabidopsis*, generate isomorphic sperm cells and therefore fusion of sperm cell appears to be random, either with the egg or the central cell (Berger et al., 2008; Ingouff et al., 2009; Liu et al., 2010). Some reports suggest that fertilization of the egg cell is preferred, which was demonstrated, for example, in mutants of *CYCLIN DEPENDENT KINASE A1* (*CDKA;1*), which generate only one sperm-like germ cell (Iwakawa et al., 2006; Nowack et al., 2006). Experiments with photo-labeled sperm cells have demonstrated that there is no preference for either female gamete (Hamamura et al., 2011). The differentiation into two equal sperm cells depends on the activity of the MYB transcription factor DUO POLLEN 1 (DUO1), which is required for correct male germ cell differentiation by regulating key genes essential for fertilization such as *GAMETE EXPRESSED 2* (*GEX2*) and *GENERATIVE CELL SPECIFIC 1* (*GCS1*), also known as *HAPLESS 2* (*HAP2*) (Brownfield et al., 2009). *GEX2* encodes a single-pass transmembrane protein with filamin repeats exposed to the extracellular space. GEX2 localizes to the sperm cell plasma membrane and contains extracellular immunoglobulin-like domains, similar to gamete interaction factors reported in algae and mammals (Misamore et al., 2003; Inoue et al., 2005). In the presence of GEX2 the two gametes adhere to the egg and central cell. *gex2* mutant sperm cells show reduced adhesion to female gametes, likely causing cell fusion failure (Mori et al., 2014). GCS1/HAP2 is another factor required for gamete interaction in *Arabidopsis*. After pollen tube burst, both sperm cells of *gcs1/hap2* loss of function mutants remain at the fusion site and fail to fuse with female gametes, leading to the attraction of additional pollen tubes (polytubey). It was further shown that in the absence of the potential fusogen GCS1/HAP2, attachment of male to the female gamete occurs but no membrane fusion is visible, implying that the protein mediates membrane fusion as a component of signaling events, or more likely that it is directly involved in the fusion event (Wong and Johnson, 2010; Mori et al., 2014). GCS1/HAP2 is a conserved protein and has been identified in genomes of all major eukaryotic taxa except fungi. *Gcs1/hap2* mutants in protozoan and algal gametes result in fusion failure, suggesting that this protein is required for a common mechanism of membrane fusion in eukaryotes (Mori et al., 2006; Hirai et al., 2008; Liu et al., 2008; Steele and Dana, 2009; Wong and Johnson, 2010). Upon sperm cell arrival at the gamete fusion site (Figure 2B) the egg cell starts to secrete small cysteine-rich proteins of the EGG CELL 1 (EC1) family. EC1 leads to the relocalization of HAP2/GCS1 from the endomembrane system to the sperm cell plasma membrane and thus activates sperm cells enabling them to fuse with the female gametes (Sprunck et al., 2012). The egg cell appears to require activation itself and calcium may play a key role in this process; this is indicated by a single strong Ca^2+^_cyto_ transient in the egg cell associated with pollen tube burst and sperm delivery (Denninger et al., 2014), which thus precedes EC1 secretion.

The relocalization of a transmembrane fusogen was also described for the mammalian-specific fusogen IZUMO1 (Inoue et al., 2005). Female components, which are directly involved in gamete fusion are so far unknown in higher plants. In mammals, CD9-like membrane spanning proteins of the tetraspanin family are located at the plasma membrane of eggs and were shown to be required for gamete fusion (Kaji et al., 2000; Le Naour et al., 2000; Miyado et al., 2000). In *Arabidopsis* the conserved tetraspanin family consists of 17 members. While TET11 and especially TET12 are located at the surface of sperm cells and reach high concentrations in the membrane region connecting both sperm cells, TET9 appears at the surface of female gametophyte cells including the egg and central cell (Boavida et al., 2013). *Arabidopsis* tetraspanins were shown to form homo- and heterodimers, but so far functional studies are missing. However, their presence at the surface of plant gametes and structural homology to mammalian CD9-like proteins suggest that they may possess a similar role during gamete interaction.

### Degradation of fusogens and prevention of polyspermy

In general polyspermy blocks prevent multiple fertilization events that would otherwise lead to abnormal development or even embryo lethality, and thus reproductive failure. In *Chlamydomonas* FUS1, a single-pass transmembrane protein with a high similarity to prokaryotic invasion and adhesion molecules, mediates membrane fusion (Ferris et al., 1996; Misamore et al., 2003). In *Chlamydomonas* both GCS1/HAP2 and FUS1 are rapidly degraded after cell fusion, resulting in a fast membrane block to prevent polygamy (Misamore et al., 2003; Liu et al., 2010). In mammals, it was recently shown that IZUMO1 is recognized by the GPI-anchored protein JUNO on the egg cell surface. Rapid degradation of JUNO after fertilization suggests an additional mechanism for membrane block to prevent polyspermy (Bianchi et al., 2014).

In *Arabidopsis* it was shown that the polyspermy block only functions in the egg cell and not in the central cell, which is capable of fusing with more than one sperm cell, as demonstrated in the *tetraspore* (*tes*) mutant. This mutant produces more than one sperm pair, which is released simultaneously at the gamete fusion site. After fertilization, polyploidy resulting from multiple fertilization events was observed in the developing endosperm, but not in the embryo (Scott et al., 2008). The cause of the egg cell-specific fast block to polyspermy is unclear. *In vitro* fertilization experiments with maize egg and sperm cells have shown that cell wall material is detectable already within 30 sec after fusion (Kranz et al., 1995) and thus may prevent further gametic membrane interactions. Additionally, a quick block to polyspermy may also depend on the degradation of fusogens as described above. Calcium may play a role in immediate signaling of successful plasmogamy and release of cell wall material as an extended Ca^2+^_cyto_ transient is observed in the egg cell associated with successful gamete fusion (Denninger et al., 2014). However, the precise cellular function of calcium signaling during gamete interaction is currently unclear and will require further experimentation.

Another way to prevent polyspermy is the deactivation of pollen tube guidance and the activation of repelling mechanisms. In *Arabidopsis* usually only a single pollen tube is guided inside the ovule to execute double fertilization. After unsuccessful fertilization events, for example by failure of cell-cell fusion in *gcs1*/*hap2, duo1, duo3, gex2, cdka;1*, or *ec1-RNAi* gametes (Beale et al., 2012; Kasahara et al., 2012; Sprunck et al., 2012; Maruyama et al., 2013; Mori et al., 2014), secondary pollen tubes are attracted by the remaining synergid cell by a process named as polytubey. This process is delayed by a couple of hours (Kasahara et al., 2012), suggesting that pollen tube repellents are released upon sperm cell discharge and require degradation until additional pollen tubes can be attracted by the remaining synergid cell. After successful fertilization this cell quickly disintegrates, but remains viable for significantly longer times upon fertilization failure (Beale et al., 2012). A recent report showed that both female gametes independently control successful fertilization thus maximizing reproductive success (Maruyama et al., 2013). The key to prevent polytubey is the quick degeneration of the 2nd synergid cell. In order to investigate its death, it was recently reported that an Ethylene-Insensitive (EIN3-EIN2)/Ethylene-Insensitive3-like2 (EIL2)-dependent, ethylene-response cascade is activated after fertilization. Its artificial activation results in premature synergid cell disintegration and thus a block to pollen tube attraction (Völz et al., 2013). The degeneration of the 2nd synergid cell thus leads to the stop of attractant secretion and ultimately prevents polyspermy.

## Activation of seed development

Both female gametes need to be fertilized to produce viable progeny. Although equipped with the genetic repertoire to generate every cell type (in the case of the fertilized egg cell) or a number of highly specialized cell types (in the case of the fertilized central cell), both female gametes appear in an arrested state until activated through fertilization. In contrast, parthenogenetic egg cells do not arrest and initiate cell division without fertilization. The central cell, however, requires fertilization in most plant species, even in those containing parthenogenetic egg cells. Its activation is closely related to seed development as both parthenogenetic embryogenesis and seed development arrest at an early stage without central cell fertilization (Koltunow and Grossniklaus, 2003; Barcaccia and Albertini, 2013). Recent reports in *Arabidopsis* confirm intensive cross-talk between endosperm and embryo as well as between endosperm and seed coat shortly after fertilization (Costa et al., 2014; Figueiredo and Köhler, 2014). It was further shown that the zygotic genome is activated shortly after fertilization in this species and both maternal and paternal genomes contribute equally to the transcriptome of the early embryo (Nodine and Bartel, 2012). Research in the last two decades has discovered many differences in epigenetic modification between male and female genomes, which lead to variations in expression profiles between their genes before and after fertilization. Polycomb group genes and RNA silencing mechanisms play a major role in these processes, but will not be considered here in more detail as excellent reviews can be found elsewhere (e.g., Van Ex et al., 2011; Gehring, 2013).

It is well conceivable that sperm cells deliver factors, which activate female gametes after fusion. A transcript encoding the Interleukin-1 Receptor-Associated Kinase (IRAK)/Pelle-like kinase gene *SHORT SUSPENSOR* (*SSP*), which was shown to be delivered by sperm cells, becomes translated in the zygote and acts in the YODA (YDA) MAPK pathway during zygote elongation (Bayer et al., 2009). In the zygote, the regulatory network activated by SSP-YDA is yet unknown. Activation of the cell cycle might also represent a key mechanism for the activation and progression of seed development. However, *cdka;1* mutant single sperm-like germ cells, defective in a master cell cycle regulator are capable of fertilizing egg cells and activating the embryonic program (Iwakawa et al., 2006; Nowack et al., 2006). Although the mutant *cdka;1* central cell showed mitotic divisions upon egg cell fertilization, it appeared mostly unfertilized, and endosperm proliferation and thereby seed development stopped after a certain time point. This finding suggested a positive proliferation signal from the zygote leading to cell cycle activation in the central cell. However, occasionally two *cdka;1* sperm cells are delivered to the gamete fusion site leading to cell-cell fusion of both female gametes with one *cdka;1* mutant sperm cell each. It was further reported that fusion between nuclei of sperm and central cell fails. The failure of karyogamy in the central cell prevents incorporation of the paternal genome, impairs endosperm development and causes seed abortion. This and the above findings using pathenogenetic species imply that the paternal genome plays an essential role during early seed development and that sperm cell factors are also required to activate central cell development (Aw et al., 2010). In summary, very little is known about the molecular mechanisms activating both female gametes that lead to the initiation of seed development.

## Conclusions

Efficient and successful fertilization of all developed ovules is a key to reproductive success and essential for high crop yield. Using the model plant *Arabidopsis* tremendous progress has been made in the past couple of years to understand the underlying cellular and molecular mechanisms that regulate pollen tube growth and guidance, sperm delivery and gamete interaction resulting in blocks to polytubey and polyspermy. Little is known about the activation of gametes and thus seed development immediately after fertilization. Many of the processes described above involve conserved mechanisms and proteins. Some of these proteins are highly polymorphic and species-specific, allowing female flower organs to discriminate self from alien pollen grains/pollen tubes to avoid reproductive failure after pollination and fertilization with incompatible gametophytes and gametes, respectively. In summary, we have learned that species-specific or even ecotype-specific molecules and plant family-specific mechanisms are required during compatible interactions. These are used by papilla cells to control pollen germination, by transmitting tract cells during pollen tube growth and by the ovule and female gametophyte cells during the last steps of pollen tube journey. Moreover, even sperm cell discharge is regulated in a species-preferential manner. Whether gamete interactions depend on species-specific molecules remains to be shown. Up to now the final processes of fertilization seem to involve partly conserved proteins even from lower to higher eukaryotes. The knowledge generated can now be used to investigate, for example, speciation mechanisms or can be applied to overcome hybridization barriers between species. Initial attempts enabling ovules to attract pollen tubes from unrelated plant families have been successful (Márton et al., 2012). However, as outlined above double fertilization mechanisms are very complex and regulated at multiple levels, and it will be a challenge to overcome all steps simultaneously allowing wide hybridization between plant species that presently cannot be crossed. A major challenge for the near future is to understand fertilization mechanisms also in crop plants, especially in the grasses, which represent the most economically important plant family. Maize was suggested as a grass and crop model to investigate these processes (Dresselhaus et al., 2011), but transformation difficulties, the low number of available insertion mutants, the requirement of sufficient greenhouse space and especially technical problems to visualize the fertilization process *in vivo* still limit its utilization for reproduction biologists. Concerted efforts are now required to understand the molecular mechanisms of double fertilization in crop plants, which significantly differ from *Arabidopsis* in both reproductive structures and genetic repertoire.

### Conflict of interest statement

The authors declare that the research was conducted in the absence of any commercial or financial relationships that could be construed as a potential conflict of interest.
