# Supplementary material for: The beginning of a seed: regulatory mechanisms of double fertilization
Source: Front Plant Sci. 2014 Sep 11;5:452. doi: 10.3389/fpls.2014.00452 (PMC4160995; doi:10.3389/fpls.2014.00452)
Supplement: Supplementary file 3 [file Presentation1.PDF]

SUPPLEMENTAL MATERIAL

Movie S1. 3D-reconstruction and animation of an *Arabidopsis* ovule from cross sections of a pistil. >150 successive ultra-thin sections were made from a fixed and embedded pistil using a microtome. After contrast staining, sections were imaged at a microscope and aligned using the 3D-visualization software Amira®. The most prominent cell components of the ovule, funiculus (petrol), outer and inner integuments (blue, purple), nucellus (red) and vacuoles and nuclei of central cell (yellow), egg cell (blue) and two synergid cells (red and green) were manually highlighted. This movie shows a cross section journey through a pistil together with a developing 3D-reconstruction of an ovule. See Figure 1 for details.

Movie S2. Rotation of a 3D-reconstruction of the same ovule as shown in Movie S1 to visualize the ovule from different angles. The funiculus is shown in petrol, the outer and inner integument are shown in blue and purple, and the nucellus in red. The vacuole and the nucleus of the central cell are shown in light yellow and yellow, respectively, and the egg cell vacuole in light blue and the nucleus in blue. The two synergid cells are color coded in red and green, respectively. See Figure 1 for details.
